# Unraveling the Epigenetic Landscape: Insights into Parkinson’s Disease, Amyotrophic Lateral Sclerosis, and Multiple Sclerosis

**DOI:** 10.3390/brainsci14060553

**Published:** 2024-05-29

**Authors:** Pierpaolo Di Martino, Valentina Marcozzi, Sandra Bibbò, Barbara Ghinassi, Angela Di Baldassarre, Giulia Gaggi, Andrea Di Credico

**Affiliations:** 1 Department of Medicine and Aging Sciences, G. D’Annunzio University of Chieti-Pescara, 66100 Chieti, Italy; pierpaolo.dimartino@studenti.unich.it (P.D.M.); valentina.marcozzi@studenti.unich.it (V.M.); sandra.bibbo@unich.it (S.B.); b.ghinassi@unich.it (B.G.); a.dibaldassarre@unich.it (A.D.B.); andrea.dicredico@unich.it (A.D.C.); 2 Cell Reprogramming and Differentiation Lab, G. D’Annunzio University of Chieti-Pescara, 66100 Chieti, Italy; 3 UdA-Tech Lab, G. D’Annunzio University of Chieti-Pescara, 66100 Chieti, Italy

**Keywords:** epigenetics, neurodegenerative diseases, Parkinson’s disease, amyotrophic lateral sclerosis, multiple sclerosis, DNA methylation, DNA histon modifications, miRNAs

## Abstract

Parkinson’s disease (PD), multiple sclerosis (MS), and amyotrophic lateral sclerosis (ALS) are examples of neurodegenerative movement disorders (NMDs), which are defined by a gradual loss of motor function that is frequently accompanied by cognitive decline. Although genetic abnormalities have long been acknowledged as significant factors, new research indicates that epigenetic alterations are crucial for the initiation and development of disease. This review delves into the complex interactions that exist between the pathophysiology of NMDs and epigenetic mechanisms such DNA methylation, histone modifications, and non-coding RNAs. Here, we examine how these epigenetic changes could affect protein aggregation, neuroinflammation, and gene expression patterns, thereby influencing the viability and functionality of neurons. Through the clarification of the epigenetic terrain underpinning neurodegenerative movement disorders, this review seeks to enhance comprehension of the underlying mechanisms of the illness and augment the creation of innovative therapeutic strategies.

## 1. Introduction

Neurodegenerative movement disorders (NMDs) are marked by neuronal degeneration, often accompanied by abnormal protein aggregate buildup in the brain, leading to motor symptoms and cognitive decline [1,2,3,4,5]. This category encompasses various diseases, including Parkinson’s disease (PD), amyotrophic lateral sclerosis (ALS), and multiple sclerosis (MS), among others. While recent advancements have partially clarified the biological mechanisms underlying the onset and progression of these diverse diseases, a complete understanding of NMDs remains elusive [6].

**Figure 1:**
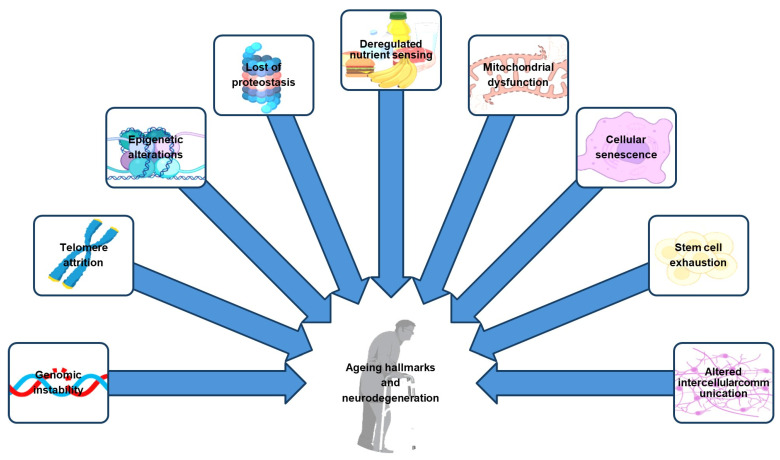
The interconnection between epigenetic, genetic, biological, and environmental factors and the onset of neurodegenerative diseases.

In recent decades, genetics and epigenetics have led to a surge in neuroscience studies aiming to understand the intricate interplay of the physiological and pathological mechanisms governing neurodevelopment and neurodegeneration. This approach led to the identification of various epigenetic modifications, such as DNA methylation, hydroxymethylation, histone modification, histone variants, and noncoding RNA [7]. These modifications dynamically regulate gene expression, playing crucial roles in shaping the neuronal landscape (Figure 1). The intersection of epigenetics with neuronal cells, known as “neuroepigenetics,” has become an area of intensified focus. Investigating the interrelationship between these epigenetic mechanisms and the disruption of neuronal function is crucial in unraveling the pathogenesis of neurodegenerative disorders [6,7]. The nuanced exploration of neuroepigenetics not only provides insights into the molecular underpinnings of neuronal development and degeneration but also illuminates potential therapeutic strategies targeting epigenetic dysregulations. This holistic approach, bridging genetics, epigenetics, and neuroscience, presents a comprehensive framework for understanding and potentially intervening in the complex processes governing brain health across the lifespan [6].

This narrative review aims to summarize recent advances in neuroepigenetic research, focusing on DNA methylation and histone changes underlying PD, ALS, and MS onset. Additionally, it examines the link between the aberrant expression of miRNAs and the onset or progression of these NMDs. The literature research was conducted using the following databases: SCOPUS, PUBMED, and MEDLINE. We considered the most important contributions in the field of epigenetic regulation in ALS, PD, and MS, considering the most updated and/or fundamental research works in the field, in order to provide the most comprehensive overview of the topic.

### 1.1. Epigenetics

Epigenetics refers to the investigation of inheritable alterations in gene expression or cellular phenotype that occur without changes to the underlying DNA sequence. These alterations can be influenced by diverse factors such as environmental cues, chemical endocrine disruptors, lifestyle choices, developmental stages, and disease conditions [8,9,10,11,12]. Epigenetic mechanisms encompass DNA methylation, histone modifications, chromatin remodeling, and regulation by non-coding RNA, all of which can regulate gene activation or repression without altering the DNA sequence itself [13]. These modifications are vital for governing gene expression, playing critical roles in normal development, cellular differentiation, and the preservation of cellular identity. The disruption of epigenetic processes has been implicated in various diseases, including neurodegenerative movement disorders (NMDs) [6,14]. In the following paragraphs, we will delve into some of these epigenetic alterations and explore their associations with the initiation or progression of NMDs.

### 1.2. DNA Methylation and Hydroxymethylation

In mammals, DNA methylation entails the transfer of methyl groups (CH3) from S-adenosyl-L-methionine (SAM), the universal donor, to cytosines, predominantly occurring within the dinucleotide context of 5′-CpG-3′ [15]. While CpG dinucleotides represent only 1% of the mammalian genome, they tend to aggregate in CpG-rich regions, termed CpG islands (CGIs), typically positioned near the transcription start sites (TSSs) of approximately 70% of human protein-coding genes [15]. The addition of methyl groups induces conformational changes in the DNA structure, impeding the recognition of methylated DNA by various transcription factors, particularly when situated in the gene promoter region, resulting in transcriptional repression [16]. DNA methylation is catalyzed by a set of proteins known as DNA methyltransferases (DNMTs), which can be classified into two groups: those responsible for de novo methylation and those responsible for methylation maintenance. For instance, DNMT1 is engaged in upholding DNA methylation patterns by attaching methyl groups to newly synthesized DNA strands during replication. Conversely, de novo DNA methylation is orchestrated by DNMT3a and DNMT3b, which add methyl groups to previously unmethylated cytosines [17]. The epigenetic significance of 5-methyl cytosine (5mC) has been extensively investigated and found to be integral to various cellular processes and functions, including developmental gene regulation, differentiation, and genomic imprinting [18]. Notably, Giallongo et al. elucidated the crucial role of DNA methylation status in modulating brain function, particularly synaptic plasticity and memory processes [19].

**Figure 2:**
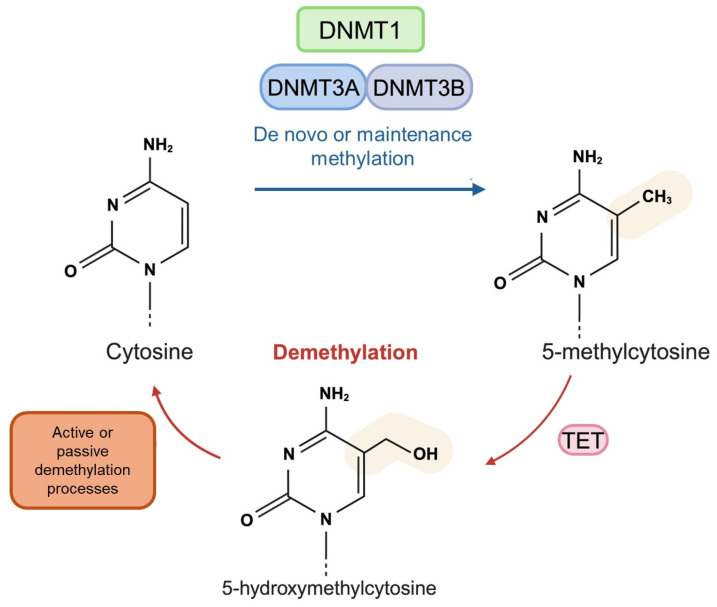
**Schematic overview of DNA methylation and demethylation processes.** DNA methyltransferases (DNMT1, DNMT3A/3B) catalyze the methylation of cytosine to 5-methylcytosine, whereas the TET proteins catalyze the hydroxymethylation of 5-methylcytosine to 5-hydroxymethylcytosine. Finally, the conversion into an unmethylated cytosine is due to the activation of active or passive demethylation processes.

On the other hand, DNA hydroxymethylation is a recently discovered DNA modification, characterized by the substitution of a hydrogen atom (H) at the C5-position in cytosine with a hydroxymethyl group (OH), resulting in the formation of 5-hydroxymethylcytosine (5hmC) [18]. The transformation of 5mC to 5hmC is facilitated by the Ten–Eleven Translocation (TET) family proteins, representing the initial phase in the DNA demethylation process, as 5hmC is swiftly converted into unmethylated cytosine [20]. Consequently, the presence of 5hmC is often transitory. Nevertheless, recent research has revealed that certain genomic regions exhibit consistent hydroxymethylation, particularly in the brain and central nervous system, indicating a fundamental role of 5hmC in the functionality, development, and pathogenesis of neuronal cells. Indeed, unlike DNA methylation, DNA hydroxymethylation is commonly linked with gene expression [21,22]. DNA methylation and demethylation processes are summarized in Figure 2.

### 1.3. Histon Modifications

**Figure 3:**
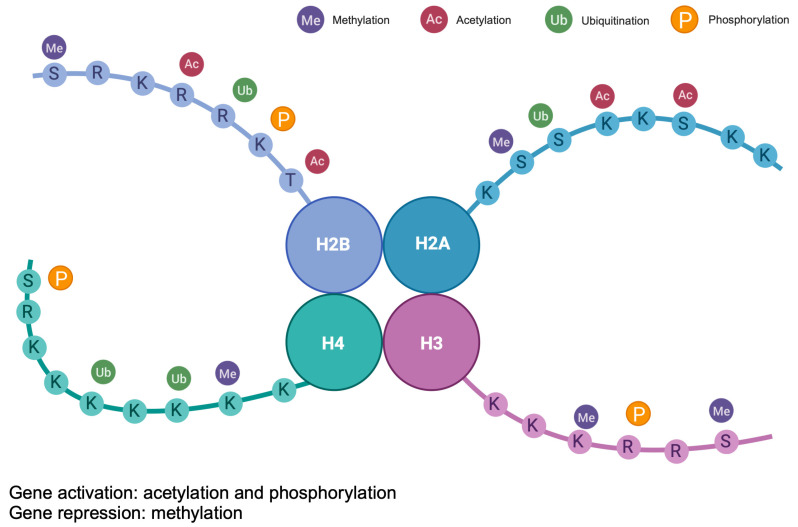
**Post-translational modifications of histones H2A, H2B, H3, and H4.** The figure shows the four major modifications: methylation, acetylation, ubiquitination, and phosphorylation.

Histone modifications encompass the biochemical changes occurring on histone proteins that contribute to the formation of chromatin [23] (Figure 3). These proteins possess long, flexible tails extending from the nucleosome core, which are susceptible to various chemical alterations like acetylation, methylation, phosphorylation, ubiquitination, SUMOylation, and ADP-ribosylation [23]. These modifications influence histone–DNA interactions and the recruitment of other proteins to chromatin, thereby impacting gene transcription, DNA repair, replication, and cell cycle progression. Consequently, histone modifications serve as a vital mechanism for regulating chromatin dynamics and gene expression patterns in response to developmental cues, environmental signals, and cellular stresses [24], playing a significant role in the epigenetic regulation of gene expression, and are implicated in various diseases, including neurological disorders [25].

Among these modifications, histone acetylation is notable and well-understood through the activities of histone acetyltransferases (HATs) and histone deacetylases (HDACs). HATs transfer an acetyl group to the ε-amino group of lysine side chains of histones, neutralizing the lysine’s positive charge. This action weakens histone–DNA interactions, facilitating gene expression by enabling the binding of the transcriptional machinery to the promoter region. Conversely, HDACs have an opposing effect, erasing lysine acetylation and restoring the positive charge. This potentially stabilizes chromatin architecture and represses transcription [26,27].

### 1.4. miRNA Regulation

**Figure 4:**
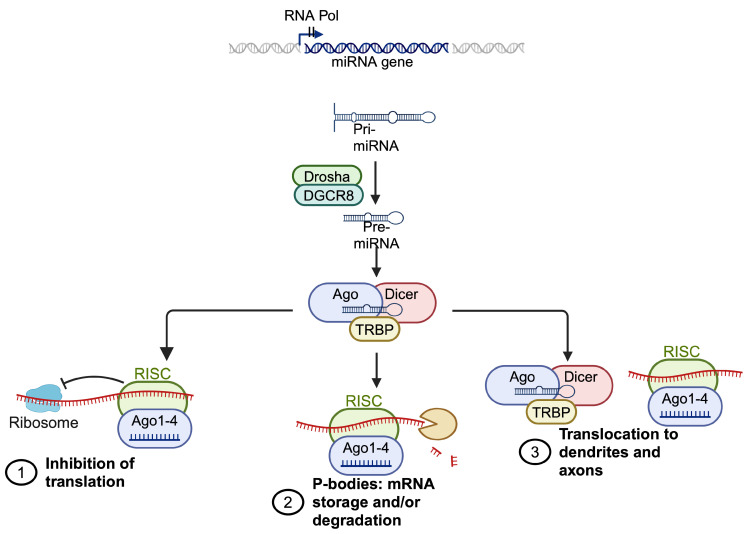
**Schematic overview of miRNA biosynthesis and action mechanisms.** pri-miRNAs are transcribed from miRNA genes encoded in exonic, intronic, or intergenic regions, and they are subsequently processed by Drosha/DGCR8 into pre-miRNAs. Once exported into the cytoplasm, pre-miRNAs are cleaved by Dicer cleavage and unwound via Argonaute (AGO) and loaded into the RNA-induced silencing complex (RISC) via TRBP. The binding of target mRNAs to miRNAs in RISC is followed by the inhibition of translation and/or mRNA degradation within p-bodies in the cytosol. The transport of RLC and RISC into the dendritic and axonal compartments occurs via a still unknown mechanism.

Non-coding RNAs (ncRNAs) fulfill a variety of functions in gene expression regulation and epigenetic processes (Figure 4). While much emphasis has traditionally been placed on protein-coding genes, it has become increasingly apparent that the majority of the genome undergoes transcription into ncRNAs, which do not code for proteins but are crucial for numerous cellular functions, including epigenetic regulation [28]. Based on their length or structure, ncRNAs can be categorized into long non-coding RNAs (lncRNAs), short non-coding RNAs (siRNAs), and circular RNAs. Studies have revealed that lncRNAs can serve as scaffolds, recruiting chromatin-modifying complexes to specific genomic loci, thereby influencing chromatin structure and gene expression [28]. For instance, lncRNAs like *TARID* and *TETILA* recruit TET proteins to the promoter region of target genes, leading to locus demethylation [29,30]. Conversely, *ecCEBPA* interacts with DNMT1, inhibiting its catalytic activity and preventing *CEBPA* methylation, a pivotal gene in myelopoiesis and granulopoiesis [31]. Additionally, short non-coding RNAs such as siRNAs and microRNAs (miRNAs) take part in RNA interference (RNAi) pathways, which can induce transcriptional gene silencing via heterochromatin formation or post-transcriptional gene silencing through mRNA degradation or the inhibition of translation [32,33]. Furthermore, certain ncRNAs function as sponges for miRNAs, sequestering them and hindering their targeting of mRNA transcripts. By modulating miRNA activity, these ncRNAs indirectly influence epigenetic regulation and gene expression [34]. Overall, ncRNAs exhibit diverse and critical roles in epigenetic regulation, contributing to the dynamic modulation of gene expression and cellular functions [28]. Understanding the functions and mechanisms of ncRNAs in epigenetic regulation is essential for unraveling the complexity of gene regulatory networks and their implications in health and disease. 

## 2. Parkinson’s Disease

PD is a progressive neurodegenerative condition that encompasses a variety of factors contributing to its onset and progression, including genetic, environmental, and epigenetic elements and polymorphisms [35,36,37,38,39]. The hallmark of PD involves the degeneration of dopaminergic neurons in the substantia nigra pars compacta (SNpc) [36,40]. Despite extensive investigation, many of the pathogenic mechanisms underlying PD remain unclear [41]. Various molecular mechanisms have been proposed to contribute to PD development, including mitochondrial dysfunction, α-synuclein (α-syn) misfolding, accumulation, and aggregation, oxidative stress, and impaired protein clearance [35].

α-syn, a presynaptic neuronal protein, plays diverse roles in vesicle transport, neurotransmitter release, and other functions, although its complete functions are not yet understood [35]. Studies employing α-syn knockout mice suggest that PD results from a gain of toxic function, wherein overexpression, mutations, and misfolding lead to the formation of proteinaceous cytoplasmic inclusions called Lewy bodies (LBs), compromising neuronal health [41]. These LBs, mainly comprised of misfolded α-syn, along with other significant protein-based aggregates, are termed Lewy neurites (LNs), with research suggesting that LBs may evolve from LNs [42].

Under normal conditions, α-syn exists in three forms: unfolded monomers, membrane-bound species, and tetramers. Disruption of this balance leads to the misfolding and aggregation of α-syn into oligomers, amyloid fibrils, and Lewy bodies [43]. Recent studies highlight the role of neuromelanin (NM) in Lewy bodies, with NM being a pigment derived from catecholamines found in human dopaminergic and noradrenergic neurons. These neurons are subject to oxidative stress due to the catecholamine metabolism, elevated unsaturated fatty acids, iron (III) ions, and insufficient mechanisms to counteract damage from reactive oxygen species (ROS) [44]. NM, acting as a metal chelator, exhibits both neuroprotective and neurodegenerative properties, with its effectiveness determined by the balance between iron and NM [45].

PD is categorized into familial PD, associated with genetic mutations, and sporadic PD, resulting from interactions between environmental and lifestyle factors [36]. Both genetic and environmental factors play critical roles in PD pathophysiology [46]. Several genetic mutations underlie PD, including the α-syn coding gene (*SNCA*), Parkin (*PRKN*), human leucine-rich repeat kinase 2 (*LRRK2*), and glucocerebrosidase (*GBA*) [35]. Environmental risk factors for PD include exposure to heavy metals, pesticides, head injuries, dairy product consumption, and type 2 diabetes [35,46]. Conversely, protective factors include coffee and tea consumption [35,47]. Also, epigenetic alterations can play a significant role in the pathogenesis of PD through diverse mechanisms, including DNA methylation, histone modifications, and miRNAs [36].

### 2.1. DNA Methylation and Hydroxymethylation in PD

Genome-wide analyses have revealed dysregulated CpG DNA methylation patterns in both the brain and blood of PD patients, suggesting a potential systemic impact by both gene gain and loss of function [36]. Notably, PD patients exhibit reduced methylation levels in *SNCA* promoter and first-intron regions, which harbor transcription-factor-binding sites, indicating that alterations in DNA methylation may influence α-syn expression [48]. The resultant increase in α-syn levels due to epigenetic modifications could contribute to the accumulation of misfolded proteins, mitochondrial dysfunction, reactive oxygen species (ROS) generation, proteasome dysfunction, and the sequestration of transcription factors, ultimately leading to general abnormal gene expression [49,50]. Additionally, α-syn has been found to sequester DNMT1 in neurons, further impacting the balance of DNA methylation in the genome [51].

Furthermore, in addition to the aberrant DNA methylation of α-syn, Rasheed et al. also reported hypomethylation of the tumor necrosis factor alpha (*TNFα*)-promoter gene in the SNpc compared to the cortex of PD patients, highlighting the potential susceptibility of SNpc neurons in PD pathogenesis due to increased proinflammatory cytokine expression [52,53].

Additional investigations have identified modifications in the DNA methylation profile of other genes implicated in PD pathogenesis. These include the hypomethylation of Nitric Oxide Synthase 2 (*NOS2*), adenosine A2A receptor (*ADORA2A*), Cytochrome P450 Family 2 Subfamily E Member 1 (*CYP2E1*), and the hypermethylation of dopamine transporter (*DAT*), suggesting a pivotal role of DNA methylation in the onset or progression of PD [54,55].

More interesting, Kochmanski et al. reported that there are sex-specific PD-associated genes that showed a different gene methylation level in male and female subjects. More specifically, performing a genome-wide analysis of DNA methylation in an enriched neuronal population from the PD post-mortem parietal cortex, they reported sex-specific PD-associated methylation changes, such as Parkinsonism-associated deglycase (*PARK7*), Solute Carrier Family 17 Member 6 (*SLC17A6*), Protein Tyrosine Phosphatase Receptor Type N2 (*PTPRN2*), and nuclear receptor subfamily 4 group A member 2 (NR4A2) genes, suggesting that epigenetics could affect males and females differently in terms of PD onset and progression [56].

Very recently, Min et al. reported the presence of aberrant hydroxymethylation in the substantia nigra of PD patients, identifying 4119 differentially hydroxymethylated regions with respect to healthy donors. Subsequent analyses revealed that the altered profile in genes was involved in many signaling pathways, such as phospholipase D, cAMP, and the GTPase Rap1 [57].

### 2.2. Histone Posttranslational Modifications in PD

In PD, the specific implications of histone modifications and their underlying mechanisms remain unclear. The research showed higher levels of histone acetylation in dopaminergic neurons from PD patients compared to controls. Exposure to environmental toxins like MPP(+) and MPTP has been shown to disrupt histone acetylation levels, suggesting their involvement in PD pathology. Moreover, the accumulation of α-syn leads to H3 hypoacetylation through histone-masking, which represents a mechanism of chromatin remodeling that could impede the expression of genes crucial for cell survival [58]. For example, interactions between α-syn and histones H1 and H3 in the nucleus form stable complexes, with α-syn binding to the *PGC1α* promoter, resulting in hypoacetylation and the reduced expression of PGC1α. Given its role as a mitochondrial transcription factor, inadequate levels of PGC1α contribute to mitochondrial dysfunction and neurodegeneration, accompanied by α-syn-induced oxidative stress and the loss of dopaminergic cells [58].

Dysfunction in mitochondrial respiratory complex 1 induces histone hyperacetylation due to a decline in the NAD+/NADH ratio, leading to a compromised functioning of sirtuins, NAD+-dependent histone deacetylases [59].

An analysis of histone acetylation changes within the primary motor cortex of PD patients revealed an increase in the acetylation of histone H3 lysine 14 (H3K14) and H3 lysine 18 (H3K18), while histone H3 lysine 9 (H3K9) acetylation showed a decrease [60].

### 2.3. MicroRNA Regulation in PD

The 3′UTR sequence of *SNCA* mRNA has the ability to bind multiple miRNAs, such as miR7, miR153, and miR34b/c. These noncoding RNAs decrease the translation of SNCA mRNA in various brain regions. The decreased expression of miR7 and miR153 suggests a potential association with the elevated α-synuclein levels in PD patients [49,61]. Kim et al. reported a downregulation of miR-133b, which is specifically expressed in midbrain dopaminergic neurons, in PD patients, implying its involvement in disease onset [25]. Further studies have revealed that miR-133b targets Paired-Like Homeodomain Transcription Factor 3 (*PITX3*), a critical transcription factor crucial for the maturation and function of midbrain dopaminergic neurons, leading to its post-transcriptional downregulation [62]. PITX3, in turn, contributes to the expression of tyrosine hydroxylase (TH), the enzyme crucial for dopamine biosynthesis [3,63].

Furthermore, Zhang et al. documented an upregulation of miR-17 in the substantia nigra of a PD mouse model and in serum samples from PD patients. This miRNA targets DNMT1, resulting in the downregulation of its protein expression, and consequently leading to abnormal DNA methylation levels in PD patients [64].

## 3. Amyotrophic Lateral Sclerosis

ALS, also known as Charcot’s disease, was first observed by the eponymous scientist 150 years ago. This neurodegenerative condition is rare, with a prevalence of 10–15 cases per 100,000 individuals, and typically manifests around the age of 60, leading to fatal progression within five years of diagnosis [65]. Despite its long history, the underlying causes of ALS remain poorly understood. A defining feature of the disease is the presence of protein aggregates in the upper and lower motor neurons located in the motor cortex and spinal cord, respectively, which contribute to the gradual loss of axons from neuromuscular junctions to motor neuron cell bodies. ALS presents with both motor and cognitive impairments, including muscle wasting, spasticity, cardiac comorbidities, difficulty swallowing, respiratory insufficiency, and cognitive symptoms like speech difficulties, irritability, obsessive behavior, and depression [66,67,68,69].

Despite extensive research, the precise pathological mechanisms underlying ALS are not fully elucidated. The evidence suggests a distinction between sporadic and familial forms, linked by common primary mutations in genes such as superoxide dismutase 1 (*SOD1*), TAR DNA-Binding Protein 43 (*TARDBP*), *FUS*, and *C9orf72* [70]. These mutations contribute to the multifaceted features of ALS, resulting in disruptions to protein homeostasis, aberrant RNA metabolism, impaired DNA repair, excitotoxicity, compromised endosomal/vesicle transport, and neuroinflammation [71,72].

The SOD1 gene, long implicated in oxidative stress and the transcription of elements within the ubiquitin–proteasome system, is associated with ALS through loss-of-function mechanisms, hindering the degradation of misfolded proteins and promoting their aggregation [73]. Autophagy regulators like *C9orf72* and *OPTN* are frequently mutated in ALS. The hexanucleotide repeat expansion (GGGGCC) in the promoter region of the *C9orf72* gene is linked to toxic gain-of-function mechanisms and RNA splicing effects [74,75]. Another mechanism implicated in ALS onset involves the dysregulated RNA metabolism. Mutations in the *TARDBP* gene, encoding for the TAR DNA-binding 43 (TDP43) protein and also found in other neurodegenerative diseases like PD, are associated with impaired RNA processing, transcription, posttranscriptional modification, and microRNA biogenesis. This leads to a toxic gain of function, culminating in the aggregation of mutant proteins in the cytoplasm [76,77]. A common feature observed in ALS is glutamate excitotoxicity, stemming from the impaired reuptake transporter in glial cells or defective transport mechanisms. This results in an excessive influx of calcium ions, triggering neuronal activation, mitochondrial dysfunction, and increased oxidative stress [78]. The dysregulation of endosomal and vesicle transport, associated with mutations in *C9orf72* and *TARDBP*, further complicates ALS pathophysiology, given their critical role in endosomal trafficking [75,79]. The onset and progression of ALS likely result from a combination of these mechanisms, culminating in the loss of axonal projections of motor neurons and neuronal networks. The high susceptibility of motor neurons remains unclear, with hypotheses ranging from metabolic alterations to the expression of specific metalloproteinases and the decrease in insulin-like growth factor 2 (IGF2), affecting neuronal sprouting. Some authors have reported the primary degeneration of fast-fatigable motor units in ALS samples, although the causes remain unknown [80].

Emerging evidence suggests that lifestyle or occupational exposures may contribute to ALS etiology, with factors such as smoking, alcohol consumption, and infectious diseases potentially playing a role. These epigenetic changes may influence the onset and severity of ALS cases and could potentially serve as early markers for diagnosis and therapeutic targets [81,82,83].

### 3.1. DNA Methylation and Hydroxymethylation in ALS

Numerous studies have documented an aberrant DNA methylation profile in individuals with ALS. For instance, Figueroa-Romero observed a widespread alteration in DNA methylation and hydroxymethylation levels in post-mortem spinal tissue from ALS patients, whereas such alterations were not detected in blood samples, indicating a neuronal tissue-specific effect [84]. Particularly noteworthy is the identification of 112 genes exhibiting hyper- or hypomethylation. Many of them are associated with immune and inflammatory responses. These changes were also reflected at the gene expression level [84]. Similarly, an investigation conducted on blood cells revealed additional genes exhibiting aberrant methylation patterns, particularly those involved in metabolism and cholesterol biosynthesis [85]. Moreover, studies have implicated a correlation between Dnmt3a and the ALS phenotype. Specifically, mice lacking *Dnmt3a* displayed a reduced number of motor neurons, like *Sod1* mutant mouse models, along with compromised neuromuscular function [86]. Intriguingly, in vitro experiments have highlighted the significant role of Dnmt3b in the development of motor neurons [87].

Similarly to PD, ALS was also reported to show an aberrant hydroxymethylation profile. Ozyurt et al. reported an increase in 5hmC levels in corticospinal motor neurons (CSMN) showing the accumulation of misfolded SOD1, but these levels were decreased in CSMN that degenerated due to the aberrant accumulation of TDP-43 [88].

### 3.2. Histone Posttranslational Modifications in ALS

In the field of epigenetic modifications implicated in ALS, researchers have observed changes in the acetylation pattern of histone proteins, potentially stemming from the dysregulated expression of HDACs, which, in turn, affect gene expression. Among these, the involvement of HDAC6 in epigenetic remodeling in ALS has garnered considerable attention. HDAC6 is known for its role in deacetylating alpha-tubulin, thereby impacting vesicle transport along axons and the clearance of misfolded proteins [89,90]. Notably, studies have indicated a decline in HDAC6 expression in the later stages of the disease, correlating with the neurotoxic effects of aggregates due to impaired autophagy processes [91].

However, it is worth noting that, at the advanced stage of the disease, increased HDAC expression may be considered a potential strategy for restoring the ubiquitin–proteasome system and overall protein homeostasis [73]. The intricate involvement of HDACs in ALS progression underscores the necessity for further investigations to elucidate the precise mechanisms and potential therapeutic implications of targeting these epigenetic regulators in the context of the disease.

Similarly, alterations in histone acetylation have also been linked to the onset or progression of ALS [86]. In a cell model overexpressing FUS, FUS was found to inhibit CBP/p300 HAT activity, leading to the hypoacetylation of the cyclin D1 (CCND1) gene, which is crucial for cell cycle progression [86]. Furthermore, in an association study, ELP3, possessing HAT activity in acetylating Lysine 14 on Histone H3 (H3K14) and Lysine 8 on Histone H4 (H4K8), was implicated in motor neuron degeneration and ALS onset. These findings were corroborated in animal models [86].

### 3.3. microRNA Regulation in ALS

The dysregulation of miRNAs has been implicated in various degenerative diseases, including ALS. Figueroa-Romero identified the dysregulated expression of 90 miRNAs in post-mortem spinal cord samples from ALS patients. These miRNAs are involved in immune response regulation, cell death, and brain development, suggesting their potential involvement in ALS etiology [92]. Notably, among them, miR-155 and miR-142 target ubiliquin 2, RNA-binding protein Fox1, and reelin; alterations in these are typically associated with neurodegeneration. Other studies have revealed the upregulation of miRNAs targeting neurofilament and Gria2 genes. These alterations are linked to motor neuron degeneration [86].

TDP43 is also intricately involved in miRNA biogenesis and the metabolism, and it has been reported that, in ALS, TDP43 is redistributed from the nucleus to cytoplasm, where it forms unfunctional aggregates. In physiological conditions, TDP43 regulates the production of miR-27b-3p and miR-181-5p, which, in turn, suppress the expression of TDP43 [77]. This negative feedback is inhibited when TDP43 localizes into the cytoplasm. ALS patients showed reduced levels of miR-27b-3p and miR-181-5p and an upregulation of cytoplasmic TDP43, suggesting that the alteration in the negative feedback between miRNAs and TDP43 may have a role in the onset and progression of ALS [93].

On the other hand, recent studies have shown that dysregulation in the skeletal muscle system is also involved in the pathogenesis of ALS. MicroRNAs specifically expressed in skeletal muscles, known as myomiRs, including miR-1, miR-23a, miR-133a/b, and miR-206, play crucial roles in controlling myogenesis [94]. Particularly, miR-206, expressed in skeletal muscles under physiological conditions, contributes to the maintenance of neuromuscular junctions and synapses, regulating myoblast differentiation [95]. Additionally, it has been discovered that miR-206 downregulates the expression levels of muscular histone deacetylase 4 (HDAC4), an inhibitor of neuromuscular junction re-innervation via fibroblast growth factor binding protein 1 (FGFBP1), suggesting the role of miR-206 in promoting re-innervation post-injury [95]. Building upon these findings, additional studies have shown that all the myomiRs are dysregulated in ALS patients. In particular, miR-1 and miR-133a/b are downregulated, suggesting their role in the onset and progression of ALS [96].

## 4. Multiple Sclerosis

MS is a chronic inflammatory demyelinating disease of the central nervous system (CNS), typically afflicting adults [97]. It is characterized primarily by inflammatory processes within the brain and spinal cord, where focal lymphocytic infiltration results in myelin and axonal damage. Initially, there is a transient inflammatory response, followed by remyelination, although this process is not durably sustained. Consequently, the early stage of the disease is marked by episodes of neurological dysfunction, often accompanied by recovery. However, as the disease advances, widespread microglial activation becomes predominant, leading to extensive and chronic neurodegeneration, ultimately resulting in progressive disability accumulation. The clinical spectrum of MS encompasses motor, sensory, visual, and autonomic system impairments [98,99].

Although much of the data suggest an autoimmune origin, the precise etiology of MS remains undefined. 

Demyelinated areas in MS give rise to sclerotic plaques, focal points of inflammation characterized by oligodendrocyte destruction, astrocytosis, and axon degeneration. The reduction in trophic support from oligodendrocytes and the myelin sheath significantly contributes to neuronal and axonal damage [98,100]. The onset and progression of MS are intricate processes influenced by genetic, epigenetic, and environmental factors, each playing a distinct role in the pathogenesis of this debilitating neurological condition [101]. The multifaceted nature of MS underscores the importance of comprehensively understanding the diverse factors contributing to its onset and progression, informing the development of targeted therapeutic strategies for effective disease management.

Numerous genes have been implicated in the pathogenesis of MS, involved in immune-mediated mechanisms, signal transduction, and even vitamin D metabolism [97,101].

Beyond genetic factors, viral infections, particularly the Epstein–Barr virus (EBV), have been identified as influential elements in the onset of MS, increasing the risk of MS onset [102]. Environmental factors such as smoking, low levels of vitamin D, and exposure to environmental and microbial toxins are also recognized as pivotal contributors to the onset of MS [100,103,104]. In addition, these environmental factors may influence the plasticity of the epigenome and contribute to epigenetic dysregulation [105].

### 4.1. DNA Methylation and Hydroxymethylation in MS

Differential methylation patterns have been observed in genes associated with the regulation of autoimmune responses (*IL2RA*, *PTPN6*, and *SOCS1*) [92,93,94] and CNS function (*PADI2*, *CDKN2A*, *RUNX3*, *NEUROG1*, and *BDNF*) [92,94,95] in both whole blood and various leukocyte populations of remitting–relapsing MS patients. This suggests a significant role of epigenetics in modulating inflammatory responses in the human brain. Moreover, genes encoding myelin-related proteins were found to be aberrantly methylated in MS patients. Olsen et al. reported the increased methylation of the Myelin Oligodendrocyte Glycoprotein gene (*MOG*) in the serum of remitting–relapsing MS patients [96]. Similarly, brain tissue analysis revealed decreased methylation in the promoter region of the peptidyl arginine deiminase type 2 (*PAD2*) gene in the white matter of MS samples compared to the control group [97]. PAD2 regulates the post-translational citrullination of myelin basic protein (MBP), which is crucial for the myelinization process [97]. The excessive citrullination of MBP affects myelin integrity, leading to its breakdown. Furthermore, PAD2 was found to be upregulated before any clinical signs of demyelination, suggesting that the aberrant hypomethylation of *PAD2* could elevate *PAD2* transcript levels, resulting in increased citrullinated MBP [97].

A genome-wide analysis, examining the DNA methylation patterns of repetitive elements such as *ALU*, *LINE1*, and *SATα*, revealed significant hypermethylation in the blood samples of MS patients compared to healthy controls [98,99]. Studies have demonstrated the downregulation of Protein Tyrosine Phosphatase (*SHP1*) in the peripheral blood leukocytes of MS subjects [93].

Investigations into normal-appearing white matter (NAWM) in MS patients have identified the hypermethylation of genes such as *BCL-2-LIKE PROTEIN 2* and *NDRG1*, involved in oligodendrocyte survival and neuronal activity, resulting in reduced protein expression compared to controls [100]. Additionally, hypermethylation was observed in genes encoding oligodendrocyte proteins (*MBP*, *SOX8*, and *GJB1*), while hypomethylation occurred in Cathepsin Z (*CTSZ*) and legumain (*LGMN*), leading to increased transcript expression. CTSZ and LGMN are cysteine proteases with diverse functions, including the proteolysis of myelin basic protein (MBP) [100]. Additionally, as expected from its inflammatory etiology, MS patients exhibit an aberrant methylation of *HLA* genes [101]. Moreover, changes in DNA methylation were found in other inflammatory pathways. Specifically, over 30% of MS patients exhibited hypermethylation of the promoter of the Src homology region 2 domain-containing phosphatase-1 (*SHP1*), resulting in its reduced expression [93,102]. The deficiency of SHP1 has broader implications, leading to the overactivity of signaling pathways involving STAT6, STAT1, and NFκB in MS patients. These transcription factors, known regulators of cell proliferation and survival, have been implicated in proinflammatory mechanisms, potentially contributing to the pathophysiology of MS [103]. The suppressor of cytokine signaling 1 (*SOCS1*) is also hypermethylated and its consequently reduced expression may aggravate the progression of MS through the hyperactivation of the immune-mediated response [101]. Additionally, MS patients exhibited higher methylation levels of *ICAM5*, which, when secreted in the cerebrospinal fluid, stimulates the secretion of anti-inflammatory cytokines, reducing T cell activation [104].

Regarding hydroxymethylation, Tang et al. reported TET1 and 2 levels in the spinal cord tissues of mice model of MS, altering the global level of 5hMC. This aspect may represent a critical target involved in myelin damage [106].

### 4.2. Histone Modification in MS

In the complex realm of multiple sclerosis (MS), the regulation of histone modifications emerges as a critical factor, as demonstrated by the heightened levels of histone H3 acetylation observed in oligodendrocyte-lineage cells within chronic MS lesions and in older patients. This surge in acetylation is associated with the increased expression of inhibitor genes linked to oligodendrocyte differentiation, potentially contributing to impaired remyelination in MS patients [107]. Interestingly, a contrasting pattern of significant deacetylation was identified in initial MS lesions, underscoring the delicate balance between acetylation and deacetylation processes [108].

SIRT1, a member of class III HDACs that rely on NAD+ for activity, emerges as a significant regulator in these modifications. Acting as a histone deacetylase, SIRT1 governs various cellular processes, including metabolism, aging, DNA repair, and inflammation. Notably, SIRT1 has been observed to be downregulated in MS relapses compared to controls, suggesting its potential involvement in the dynamic epigenetic landscape of MS [109].

Another crucial histone post-translational modification is methylation, notably evidenced by diminished levels of histone H3 methylation in MS lesions, including a decrease in histone H3 trimethylation (H3K4me3). This decline is attributed to reduced methyl donors in the grey matter neurons of MS patients, leading to disruptions in the mitochondrial metabolism. H3K4me3 is intricately linked to the expression of the mitochondrial electron transport chain (ETC), and the metabolite N-acetylaspartate (NAA) is associated with ETC activity in neurons, influencing neuroaxonal metabolism [110,111]. Deficiencies in these processes could anticipate neuronal deterioration in MS [110]. Moreover, NAA emerges as a pivotal factor in sustaining myelination through epigenetic mechanisms in oligodendrocytes, particularly those involving H3 methylation [108]. The intricate network of histone modifications further extends to the upregulation of PADI2 due to the hypomethylation of its promoter. PADI2, in turn, enhances the expression of PADI4, an enzyme implicated in the citrullination of H3 histone, which is responsible for initiating apoptosis in oligodendrocytes [110].

### 4.3. microRNA Regulation in MS

An analysis of microRNA (miRNA) profiles has uncovered significant changes in the expression of various miRNAs in both the brains and circulating blood of individuals diagnosed with multiple sclerosis (MS) [107]. This review focuses on the investigation of specific miRNAs, namely miRNA34a, miRNA155, and miRNA326 in brain white matter, and miR155, miR338, and miR491 in brain white matter, along with the miR17/92 cluster in whole blood. Within active MS lesions, miR34a, miR155, and miR326 show increased expression, targeting CD47, a glycoprotein widely found in oligodendrocytes (myelin) and astrocytes. CD47 plays a vital role in self-recognition and prevents phagocytosis by macrophages [112]. The upregulation of these miRNAs results in the suppression of CD47 expression, leading to myelin destruction by macrophages [112].

MicroRNA155 and microRNA326 also contribute to T-cell differentiation, particularly promoting the development of T-helper 17 (TH17) cells, which are abundant in MS lesions [107,112]. CD47 is significantly downregulated in active MS lesions compared to controls, while its expression in inactive lesions is comparable to control white matter [112]. MiR326 targets Ets1 and Foxp3, two regulators inhibiting TH17 cell differentiation [113].

White matter miRNA profiling in brain autopsies of MS patients reveals elevated levels of miR155, miR338, and miR491 compared to controls. These miRNAs target transcripts of two isoforms of the aldoketo reductase family 1 C1 and C2 (*AKR1C1* and *AKRC2*), primarily expressed in the brain. These isoforms are involved in the synthesis of neurosteroids, which play crucial roles in neural cell functions and various pathological conditions [114]. The upregulation of these miRNAs leads to a reduction in steroid-related enzymes and neurosteroid levels, including the neuroprotective allopregnanolone that binds GABA receptors [107,114,115,116].

The miR17/92 cluster, consisting of miR17, miR18a, miR19a, miR20a, miR19b1, and miR92a1, has been implicated in immune, cardiovascular, and neurodegenerative diseases, as well as normal development and aging [117]. Among these, miR17 and miR19b1 have been studied as regulators of the immune response and promoters of CD4+ T-cell functions, playing a pivotal role in TH17 cell differentiation by inhibiting IKZF4 and enhancing the PI3K-AKT-mTOR pathway by repressing PTEN [118]. Contradictory results have emerged regarding the expression of miR17/92, particularly miR17 and miR20a, in MS patients, highlighting the complex and nuanced regulatory roles of these miRNAs in the context of MS pathogenesis [119,120].

## 5. Epigenetic Therapy

For many years, the pursuit to understand the pathogenesis of neurodegenerative diseases has been relentless. Although genetic and environmental factors have been identified, effective treatment options remain limited, and they only are able to slow the progression of the diseases, while an effective cure is still elusive [121]. Increasing evidence highlights the crucial role of epigenetics in the development and progression of PD, MS, and ALS, offering a theoretical foundation for the use of pharmacological and genetic tools to modulate these neurodegenerative disorders. Indeed, since the processes leading to alterations in epigenetics are reversible, several epigenetic marks have been proposed as potential therapeutic targets to treat or delay neurodegeneration [122].

In the following sections, we summarize the most effective therapeutic tools targeting epigenetics in PD, ALS, and MS.

### 5.1. Epigenetic Therapy in PD

Epigenetic drugs targeting DNA methylases have shown promise in PD. One such drug is epigallocatechin-3-gallate (EGCG), a natural compound from green tea, which completed its Phase II clinical trial in 2009 [123]. EGCG has demonstrated neuroprotective effects in PD models and in reducing the risk and progression of PD [124]. More specifically, EGCG inhibits DNMT1 by forming multiple hydrogen bonds, preventing cytosine methylation. EGCG also mitigates α-syn toxicity by remodeling toxic α-syn fibers and redirecting their aggregation into non-toxic forms [124]. Additionally, EGCG binds with dysregulated copper ions (Cu(II)) that are strongly connected with the formation of abnormal aggregates of α-syn into a β-sheet structure, which displayed high toxicity [125]. In addition, EGCG reduced Cu(II)-induced reactive oxygen species (ROS) that are commonly associated with cellular toxicity [125]. Drug delivery studies have suggested that EGCG, when formulated with specific lipids, can cross the blood–brain barrier, enhancing its therapeutic potential for neurodegenerative diseases [126].

Other DNMT inhibitors, such as curcumin derivatives, catechins, and bioflavonoids (e.g., quercetin), discovered through high-throughput screening, or some existing drugs, like hydralazine, procainamide, and procaine, approved for other conditions, may have a potential therapeutic effect and could be repurposed for neurodegenerative diseases. RG108, another promising DNMT inhibitor, has shown effectiveness in inducing DNA demethylation without toxicity in vitro, making it a good candidate for future PD treatments. Despite these advances, most DNMT inhibitors have not yet entered clinical trials for PD, but their optimization continues to be a promising area for the development of new treatments [127].

Similarly, HDAC inhibitors have been shown to have significant neuroprotective effects. Sodium butyrate (approved by the FDA for various treatments) effectively inhibits different classes of HDACs, leading to hyperacetylation and promoter activation. It crosses the blood–brain barrier and protects dopaminergic neurons from oxidative stress and α-syn toxicity, enhancing the expression of some neurotrophic factors, like GDNF and BDNF, which are crucial for neuronal growth, survival, and synaptic plasticity [58,128]. In addition, it has been shown to upregulate the neuroprotective heat shock protein 70 (Hsp70) in rat astrocytes, reduce neuroinflammation and oxidative stress in 6-OHDA-induced PD rat models, and prevent MPTP-mediated apoptosis in human-derived SK-N-SH and rat-derived MES 23.5 cells. Sodium butyrate has also been found to restore the expression levels of DNA repair genes such as FOXM1 and BRCA2, offering neuroprotection against α-syn-mediated DNA damage. 

Trichostatin A (TSA), a natural product from streptomyces hygroscopicus, was initially an antifungal antibiotic. TSA is highly selective to HDAC3 and promotes histone H4 acetylation. It reduces α-syn neurotoxicity and early mortality in a PD drosophila model, mitigates inflammatory cytokines secreted by activated microglia, and ameliorates motor dysfunction [129]. 

Recent studies have highlighted that treatment of SH-SY5Y cells with LMK235, another HDAC inhibitor, significantly increases the expression of acetyl histone H3-K9 and H3-K14, promoting neurite outgrowth through the activation of the BMP–Smad signaling pathway, thus protecting axons from degeneration [130].

Similarly, regulation of the activity of HATs could be an alternative epigenetic therapeutic approach. 

CTPB, a selective small-molecule activator of p300/CBP, significantly activates p300 HAT activity, protecting neuronal cells from 6-OHDA-induced cell death, promoting their survival and neurite growth. Garcinol, a p300/CBP HAT inhibitor extracted from Garcinia yunnanensis, protects cells from MPP+-induced cell death. Other HAT inhibitors, such as anacardic acid and curcumin, have been shown to improve L-DOPA-induced dyskinesia in PD patients [131]. 

Recently, miRNA-based therapeutic strategies have also been developed, utilizing either miRNA mimics or an anti-miRNA to restore or to antagonize a miRNA target, respectively [127].

In this scenario, Zhou et al. found that miR-7 mimics reduced MPTP-induced dopaminergic degeneration in the striatum in a mouse model of PD, providing a neuroprotective effect. MiR-7 was shown to downregulate the NLRP3 inflammasome in the midbrain, inhibit caspase-1 activation, and reduce the proinflammatory cytokine IL-1β [132]. Another study demonstrated that miR-425 mimics attenuated necroptosis activation and dopaminergic neuron loss, improving locomotor behavior [133]. Additionally, miR-29a mimic therapy decreased cell death and ROS generation while enhancing protective enzymes in SH-SY5Y cells after MPP+ treatment [134]. The miRNA Let-7 regulated α-syn via the autophagy–lysosome pathway, and its inhibition reduced α-syn expression without affecting dopaminergic/acetylcholinergic neurons [135]. Moreover, inhibiting miR-155 reduced TNF-α-induced cell death in SH-SY5Y [136], and miR-421 inhibition protected neurons against neurotoxicity in PD cellular and animal models [137]. 

### 5.2. Epigenetic Therapy in ALS

The effectiveness of HDAC inhibitors has also been tested in ALS models. More specifically, Ryu et al. reported that the administration of 4-phenylbutryrate, starting before or shortly after the beginning of symptoms, resulted in increased survival and reduced clinical impairment in ALS mouse model [138]. Another study reported that the administration of valproic acid in *Sod1* mutant mice significantly reduced the death of motor neurons, leading to a general neuroprotection. Despite this, the treatment was only able to slightly delay the onset of motor symptoms and muscle atrophy and, more importantly, it did not increase the mean survival of mice [139]. HDAC inhibition also rescued the DNA repair response in iPSC-derived motor neurons carrying the ALS-associated FUS^P525L^ mutation [140]. Finally, the treatment of SOD-G93A transgenic ALS mice with TSA soon after the onset of motor symptoms reduced neuron death, and ameliorated muscle atrophy and neuromuscular junction denervation. More importantly, the treatment increase the mean survival duration by 18% [141].

Similarly to PD, in ALS, therapeutic strategies have been developed using antisense nucleotides (ASOs) that are designed to target and degrade a specific mRNA. This strategy is particularly effective in ALS conditions with specific gene mutations [142]. At this moment, there are several ASOs in clinical trials, such as Torfesen in patients with *SOD1* mutations. Indeed, Torfesen is designed to target mutant *SOD1*, reducing protein expression. More interestingly, patients who received ASO during the earlier disease stages showed a smaller decline in ALSFRS-R score [143]. 

### 5.3. Epigenetic Therapy in MS

In MS, different classes of epigenetic drugs have been tested, but the majority of the work focused on DNMT and HDAC inhibitors [144]. 

In the field of DNMT inhibitors, Mangano et al. demonstrated that decitabine significantly improved clinical and histological outcomes in two mouse models of MS. More specifically, decitabine raised the transcript levels of anti-inflammatory cytokines and lowered mRNA expression of pro-inflammatory mediators. The treatment also increased the proportion of circulating regulatory T cells by inducing Foxp3 expression through the demethylation of a CpG island in the *Foxp3* gene [145]. 

However, regarding HDAC inhibitors, Camelo et al. demonstrated that TSA alleviated symptoms of spinal cord inflammation, demyelination, neuronal and axonal loss, and disability in mice during the relapsing phase. It also increased the mRNA levels of genes associated with antioxidants, anti-excitotoxicity, and neuronal growth, whereas it inhibited caspase activation and downregulated pro-apoptotic E2F transcription factor pathway genes [146]. Ge et al. showed that the HDAC inhibitor Vorinostat inhibited the differentiation, maturation, and endocytosis of human CD14(+) monocyte-derived dendritic cells (DCs), reducing their ability to stimulate allogenic T cell proliferation in vitro. Vorinostat also decreased Th1 and Th17 cytokine production and reduced CNS inflammation and demyelination in a mouse model of MS [147].

The research has also explored the role of miRNAs in treating MS. Wang et al. found that miRNA-219 mimics promoted remyelination and improved motor function in animal models [148].

Junker et al. identified miRNA-34a, miRNA-155, and miRNA-326 as potential targets for reducing MS lesion activity by regulating CD47, which affects macrophage activation and myelin phagocytosis [112].

Morquette et al. observed that miRNA-223 and miRNA-27a-3p protect against glutamate toxicity in MS, suggesting potential therapeutic targets [149], whereas Zhang et al. showed that miRNA-26a modulates Th17 cytokine expression, indicating its potential for MS therapy [150].

Emerging therapies also include modulators of miRNA expression. Nanocurcumin decreased miRNA-155 and miRNA-16 in MS patients, which are involved in T cell-mediated autoimmunity [151].

## 6. Conclusions

In conclusion, the emerging understanding of epigenetic modifications has shed light on their pivotal role in the progression and onset of neurodegenerative diseases, such as PD, ALS, and MS. Epigenetic alterations can dysregulate the expression of key genes involved in dopaminergic neuron function, protein aggregation, and oxidative stress response, mitochondrial functions that induce or exacerbate neuronal damage and impair cellular homeostasis, ultimately leading to the typical motor symptoms of these motor diseases. Moreover, neuroinflammation, a common condition detected in both PD, ALS, and MS, can contribute to alterations in the epigenetic profile of the cells and may promote the progression of the diseases. In MS, additional changes in the epigenetic landscape of immune cells can promote their activation and infiltration into the central nervous system, contributing to neurodegeneration.

Overall, the identification of epigenetic modifications as key players in the pathogenesis of Parkinson’s disease, ALS, and MS holds great promise for the development of novel therapeutic strategies. Targeting epigenetic regulators could offer new therapeutic approaches for potentially slowing disease progression and improving outcomes for patients affected by these neurodegenerative disorders.
